# Plant genotype-specific modulation of *Clonostachys rosea*-mediated biocontrol of septoria tritici blotch disease in wheat

**DOI:** 10.1186/s12870-025-06620-9

**Published:** 2025-05-02

**Authors:** Sidhant Chaudhary, Mustafa Zakieh, Mukesh Dubey, Dan Funck Jensen, Laura Grenville-Briggs, Aakash Chawade, Magnus Karlsson

**Affiliations:** 1 Department of Forest Mycology and Plant Pathology, Swedish University of Agricultural Sciences, Uppsala, SE-75007 Sweden; 2 Department of Plant Breeding, Swedish University of Agricultural Sciences, Lomma, SE-23422 Sweden; 3 Department of Plant Protection Biology, Swedish University of Agricultural Sciences, Lomma, SE-23422 Sweden

**Keywords:** Biological control agent, *Clonostachys rosea*, Genome-wide association study, Integrated pest management, Septoria tritici blotch, Single nucleotide polymorphism, Winter wheat, *Zymoseptoria tritici*

## Abstract

**Background:**

Beneficial microorganisms can act as biological control agents (BCAs) directly by targeting pathogens or indirectly by enhancing the plant’s defense mechanisms against pathogens. However, efficiencies with which plants benefit from BCAs vary, potentially because of genetic variation in plants for plant-BCA compatibility. The aim of this study was to explore the genetic variation in winter wheat for modulation of *Clonostachys rosea*-mediated biocontrol of septoria tritici blotch disease caused by the fungal pathogen *Zymoseptoria tritici*.

**Results:**

In total, 202 winter wheat genotypes, including landraces and old cultivars grown from 1900 onwards in the Scandinavian countries, were tested under greenhouse-controlled conditions. Foliar spray applications of the pathogen and the fungal BCA in two treatments, i.e., *Z. tritici* (Zt) alone and *Z. tritici* along with *C. rosea* (ZtCr) were used to assess the disease progress over time. The absence and presence of *C. rosea* in Zt and ZtCr, respectively, allowed the dissection of variation for plant disease resistance and biocontrol efficacy. The study showed significant (*P* < 0.05) phenotypic variation among plant genotypes for disease progression in both Zt and ZtCr treatments. Moreover, the application of *C. rosea* resulted in a significant (*P* < 0.05) reduction in disease progression for seven genotypes and increased disease progression for eleven genotypes, indicating a plant genotype-dependent effect on the interaction between wheat, *C. rosea* and *Z. tritici*. For the phenotypic variation in disease progress and biocontrol efficacy, a genome-wide association study using a 20K single-nucleotide polymorphism (SNP) marker array was also performed. In total, five distinct SNP markers associated with disease resistance and four SNP markers associated with *C. rosea* biocontrol efficacy were identified.

**Conclusions:**

This work serves as a foundation to further characterize the genetic basis of plant-BCA interactions when inoculated with *Z. tritici*, facilitating opportunities for simultaneous breeding for disease resistance and biocontrol efficacy.

**Supplementary Information:**

The online version contains supplementary material available at 10.1186/s12870-025-06620-9.

## Background

Septoria tritici blotch (STB) caused by the fungal pathogen *Zymoseptoria tritici* is one of the major fungal foliar diseases of wheat worldwide, which can cause up to 50% yield losses during severe epidemics in Europe [1, 2]. *Zymoseptoria tritici* goes through several cycles of sexual and asexual reproduction during a growing season, resulting in repeated infection of new plants [3]. The fungus produces pseudothecia fruiting bodies that release airborne, sexually produced ascospores, while asexual fruiting bodies called pycnidia generate conidia that are dispersed mainly through rain splash [3, 4]. Currently, the main control measures include cultivation of disease-resistant wheat varieties and fungicide applications. However, because of its high evolutionary potential, *Z. tritici* can rapidly overcome plant resistance and adapt to single-target fungicides [5–7]. Therefore, new control measures are needed to complement the existing strategies in an integrated pest management (IPM) context.

One such potential measure to reduce fungicide use and the risk for fungicide-resistant pathogens is the use of microorganisms for biological disease control of STB. Biological control or biocontrol is defined as the exploitation of living organisms to combat pests and pathogens, directly or indirectly, to provide human benefits such as reduced yield loss [8]. Biological control can be further subdivided into natural and conservation biocontrol where the resident natural enemies are used to control pathogens, and classical and augmentative biocontrol where mass-reared BCAs are released into target areas [8]. Biocontrol of plant diseases is an attractive alternative to chemical control in conventional agriculture and it can also be utilized in organic agricultural practices. In Europe, there are strong political incentives to develop biological control as an important component for sustainable plant production within IPM strategies. For example, the European Green Deal states that the use of synthetic chemical pesticides should be reduced by 50% by 2030, and biological control is specifically mentioned by the European Commission [9] in its proposal for a new regulation on sustainable use of plant protection products.

One such BCA is *Clonostachys rosea*, which is an ascomycete fungus with a generalist lifestyle including saprotrophism, plant endophytism and mycoparasitism [10, 11]. Certain strains of *C. rosea* can control plant diseases and are currently used for augmentative biological control [10]. Until now, *C. rosea* has been reported to exhibit biocontrol properties against more than 30 common fungal and oomycete plant pathogens, including *Pythium tracheiphilum* [12], *Alternaria* spp. [13], *Botrytis cinerea* [14], *Fusarium* spp. [15] and *Bipolaris sorokiniana* [16], on a range of crops including fruits, vegetables, pulses, cereals, oil crops and forest trees [10]. More recently, *C. rosea* strain IK726 was also shown to significantly control naturally occurring STB on one wheat genotype under field conditions [17]. Mechanistically, *C. rosea* employs different strategies during microbial interactions such as competition for space and nutrients [18], antibiosis [19, 20], induction of plant defense responses [21, 22] and direct parasitism [10, 23]. Certain strains of *C. rosea* can also positively and negatively modify the populations of soil bacteria, protozoa and fungi [24, 25].

Most work on augmentative biological control of plant diseases typically involves single or very few plant genotypes. Therefore, exploring the impact of plant genotypic differences on BCA efficacy and their potential use in plant breeding remains a challenge. Nevertheless, the limited examples that exist have shown that host plant genotypes can play important roles in the outcome of biocontrol interactions by affecting plant colonization by BCA and pathogen, plant anatomy and physiology and induction of plant defense immune responses [26]. Moraga-Suazo et al. [27] reported a differential response towards *C. rosea*-mediated biocontrol of the pitch canker pathogen *Fusarium circinatum* between *Pinus radiata* genotypes. This study also showed that the ability to activate induced systemic resistance (ISR) differed between the pine genotypes, providing indications of the underlying mechanism of the phenomenon. Similarly, Tucci et al. [28] observed differences between five tomato genotypes for enhanced ISR against the grey mold pathogen *B. cinerea* using *Trichoderma atroviride* and *Trichoderma harzianum*. Plant genotype differences for *Trichoderma*-mediated growth promotion in the absence of pathogens were also reported for sugar beet [29] and lentils [30]. Smith et al. [31] demonstrated variation among 61 tomato genotypes in their interaction with the disease-suppressive bacterium *Bacillus cereus* against the pathogen *Pythium torulosum*. More recently, Esmail et al. [32] also highlighted the role of *T. asperellum* strain T34 in inducing resistance in 198 spring wheat genotypes against wheat stripe rust. In our recent study in winter wheat, we show significant variation among plant genotypes for biocontrol of fusarium foot rot using *C. rosea* [33]. From these examples, it is evident that plant genotypes influence the ability of plants to benefit from beneficial microorganisms. Hence, it is important to consider plant genetic variation for efficient deployment of BCAs.

Breeding for plant crop variety development with enhanced compatibility with beneficial microorganisms offers an additional strategy for efficient plant protection within an IPM context. This requires an understanding of the genetic inheritance of the compatibility trait, and identification of genetic markers useful for marker-assisted breeding or genomic selection approaches. Understanding the nature of the relationship between various agronomic traits and disease resistance traits is an important step towards simultaneous breeding with minimum to no penalty on traditional breeding traits of yield, quality and relevant biotic and abiotic stress resistance. Recent advances in DNA sequencing technology have enabled large-scale genotyping-by-sequencing approaches useful for large and complex genomes such as *Triticum aestivum* [34], which can be exploited in genome-wide association studies (GWAS) of traits such as BCA compatibility.

In the current work, we hypothesized that winter wheat genotypes exhibit variation in their ability to benefit from *C. rosea*-mediated biocontrol of STB disease. The objectives of the study were to: (i) evaluate the variation among wheat genotypes for resistance to *Z. tritici* causing STB and for *C. rosea*-mediated biocontrol efficacy against STB, and (ii) perform a genome-wide association study (GWAS) to identify marker-trait associations for STB resistance and *C. rosea*-induced biocontrol efficacy, as well as to investigate whether these traits are inherited independently or together.

## Methods

### *Clonostachys rosea* formulation production and application

*Clonostachys rosea* strain IK726 initially isolated from barley roots in Denmark [35] was used in the current work. The strain was revived from a 20% glycerol conidial suspension stored at −80 °C and then maintained on potato dextrose agar media (PDA; BD Difco Laboratories, France) at 20 °C in dark conditions. For greenhouse bioassays, a formulation of *C. rosea* IK726 was prepared using a sphagnum peat and wheat bran mixture following a previously described method by Jensen et al. [36] with some modifications. Briefly, mass production of *C. rosea* IK726 was prepared from growth on a mixture of sphagnum peat, wheat bran, and water (3:5:12 w/w/w). The mixture was autoclaved twice on two successive days for 20 min. Forty grams mixture was put in reagent bottles with caps and inoculated with three agar plugs (5 mm diameter) of *C. rosea* from PDA plates. The fungus was incubated for 20 days at 20 °C with regular shaking of bottles manually to promote distribution of *C. rosea* spores. At harvest, the mixture was taken out of the bottles under sterile conditions and was air-dried for two days. Colony forming units (cfu) in the mixture were estimated using a tenfold serial dilution, grown on PDA media petri plates, and the mixture was stored in vacuum sealed bags at 4 °C until use. According to Jensen et al. [36], the formulation remains viable for up to 6 months, and in this study, it was stored for 3 months.

For foliar application of *C. rosea* in greenhouse bioassays, the formulation was adjusted to 1 × 10^7^ cfu/ml by adding sterile distilled water. The formulation in water suspension was shaken for 30 min to release fungal spores and followed by filtration through miracloth (Merck KGaA, Darmstadt, Germany) to remove larger clumps of mycelium or growth substrate. Polyoxyethylene (20) sorbitan monolaurate or Tween 20 (Sigma-Aldrich Chemie GmbH, Steinheim, Germany) was added to a final concentration of 0.1% (v/v) in the *C. rosea* suspension solution as a surfactant immediately before spraying.

### *Zymoseptoria tritici* preparation and application

*Zymoseptoria tritici* strain Alnarp 1 was used in this study, which was isolated from STB lesions on leaves of winter wheat collected in 2015 in Lomma, Sweden [37]. The strain was revived from a 50% glycerol conidial suspension stored at −80 °C and then maintained by adding 10 µl of the spore suspension in the middle of 9 cm diameter yeast malt sucrose (YMS medium) agar plates, which contained 4 g of yeast extract (Merck KGaA, Darmstadt, Germany), 4 g of malt extract (Duchefa Biochemie, Haarlem, The Netherlands) and 4 g of sucrose (VWR International, Leuven, Belgium) in 1000 ml of water [38]. After one day of growth on petri plates, the growing culture was spread using a glass spreader to the entire plate by adding one ml sterile water. Inoculated petri plates were incubated at 20 °C for ten to twelve days.

For foliar application, *Z. tritici* was harvested by adding sterile distilled water in each petri plate and scraping the mycelial surface with a sterile paint brush to release conidia and spores. The suspension was filtered through a single layer of miracloth (Merck KGaA, Darmstadt, Germany). The concentration of the filtrate was determined using an improved Neubauer hemacytometer (Hausser Scientific, Horsham, PA) and was adjusted to 1 × 10^6^ cfu/ml in the final suspension. Tween 20 (Sigma-Aldrich Chemie GmbH, Steinheim, Germany) was added to a final concentration of 0.1% (v/v) as a surfactant in the suspension before spraying.

### Bioassay experiments

#### Small-scale biocontrol efficacy screening

An initial experiment in a growth chamber with controlled conditions was performed to optimize concentrations of *Z. tritici* and *C. rosea*, to confirm STB disease development and *C. rosea* biocontrol of STB. Four winter wheat genotypes with varying susceptibility to STB were used: Nimbus (susceptible), Kask (susceptible), SW_150428 (resistant) and Festival (resistant). Four wheat seeds were sown per plastic pot (9 × 9x8 cm) in potting soil (Såjord, Hasselfors Garden AB, Sweden; NPK 14–7–15, pH 5.5–6.5) and were placed in trays. Plants were propagated at 60% relative humidity (RH) with the light period (light intensity of 300 μmol/m^2^) of 16 h at 20 °C and dark period of 8 h at 16 °C. For each genotype, six treatments were used i.e., 1.) control (mock treatment with no *C. rosea* and no *Z. tritici*), 2.) Cr 1 × 10^8^ (*C. rosea* at 1 × 10^8^ cfu/ml), 3.) Zt 1 × 10^6^ (*Z. tritici* at 1 × 10^6^ cfu/ml), 4.) Cr 1 × 10^8^ & Zt 1 × 10^6^ (*C. rosea* at 1 × 10^8^ cfu/ml and *Z. tritici* at 1 × 10^6^ cfu/ml), 5.) Cr 1 × 10^8^ & Zt 5 × 10^5^ (*C. rosea* at 1 × 10^8^ cfu/ml and *Z. tritici* at 5 × 10^5^ cfu/ml) and 6.) Cr 1 × 10^8^ & Zt 1 × 10^5^ (*C. rosea* at 1 × 10^8^ cfu/ml and *Z. tritici* at 1 × 10^5^ cfu/ml). These concentrations were chosen based on previous studies using *C. rosea* [10]. With these treatments, it was possible to observe the effect of *C. rosea, Z. tritici* and how varying levels of *Z. tritici* are controlled by *C. rosea*. Five biological replicates (five pots with four plants each) were used for each treatment in each genotype.

**Fig. 1:**
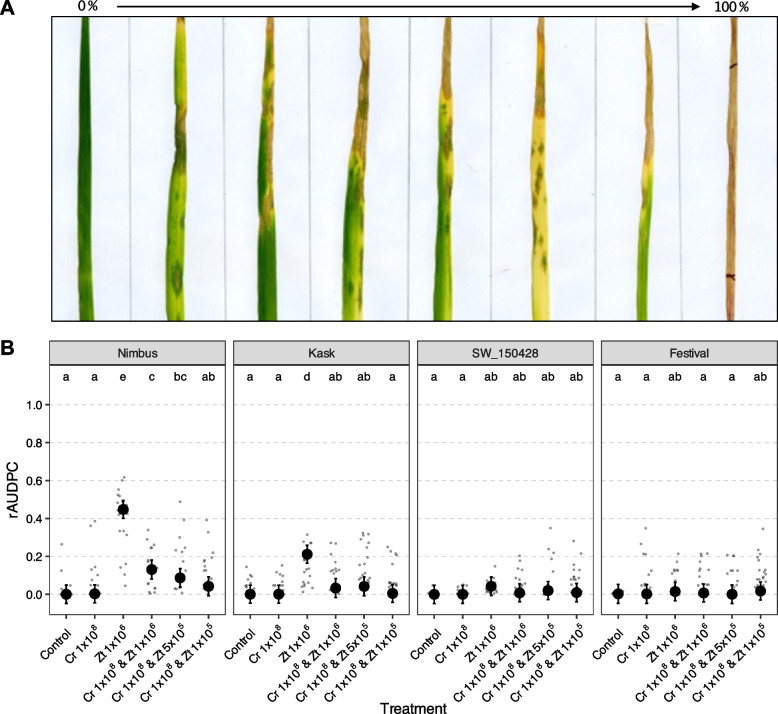
**A** Septoria tritici blotch (STB) disease severity symptoms ranging from no necrosis (left) to complete leaf necrosis (right). **B** Biological control of septoria tritici blotch disease on wheat. Four winter wheat genotypes with low (Nimbus and Kask) and high (SW_150428 and Festival) disease resistance, respectively, were inoculated with varying concentrations of the biological control agent *C. rosea* and *Z. tritici*. Relative area under disease progress curve (rAUDPC) was used as an estimate of disease severity. Grey points represent raw estimates of rAUDPC values from technical replicates. Black points represent the model estimated means and error bars represent 95% confidence intervals. Treatments not sharing the same letters indicate significant difference (*P* < 0.05) across genotypes as determined by Tukey’s post-hoc pairwise comparisons test

For *C. rosea* application, 20-day old plants were sprayed with *C. rosea* suspension (control treatment and Zt 1 × 10^6^ were sprayed with water only) until run-off using a handheld sprayer. Inoculated plants were kept under dark conditions with constant > 85% RH. After 24 h, plants were sprayed with *Z. tritici* suspension (control treatment and Cr 1 × 10^8^ were sprayed with water only) in the same manner as *C. rosea* application. Inoculated plants were kept under dark conditions with constant > 85% RH. After 48 h, the growth chamber was brought back to standard conditions of 60% RH with the light period (light intensity of 300 μmol/m^2^) of 16 h at 20 °C and dark period of 8 h at 16 °C. Percentage of necrotic leaf area was used as a proxy for disease and was visually scored from 0 to 100% with 5% interval (Fig. 1A). Disease scoring was performed on the (marked) 3rd leaf of each plant at 14, 16, 18, 20, 22, 25, 27 and 30 days post inoculation (dpi) with *Z. tritici*. Using these time points of disease scoring, the relative area under the disease progress curve (rAUDPC) in each condition was estimated as below:

$$rAUDPC= \sum_{i=1}^{n-1}\frac{{y}_{i}+{y}_{i+1}}{2} \times ({t}_{i+1}-{t}_{i})/{100 \times (t}_{n}-{t}_{1})$$

where ${y}_{i}$ is the disease score in percent at timepoint ${t}_{i}$, ${t}_{i+1}- {t}_{i}$ is the time interval between two scorings and $n$ is the total number of scoring time points. ${100 \times (t}_{n}-{t}_{1})$ is the AUDPC maximum used in the denominator to estimate the relative AUDPC.

#### Large-scale biocontrol efficacy screening

A total of 202 winter wheat genotypes were used, which comprised of landraces (n = 33), cultivars (n = 155) and other undefined genotypes from the Scandinavian countries grown between 1900 and 2012 (Supp. Table 1). The seeds were initially obtained from Nordic Genetic Resources Centre, Alnarp, Sweden, and were later multiplied in the field southern Sweden. Seeds were placed on moist filter paper in empty petri plates for four days at 4 °C in dark conditions and were then transferred to room temperature for three days for germination. Germinated seedlings were transplanted into plastic pots (9 × 9 × 8 cm) filled with peat substrate based potting soil (Yrkesplantjord, Gröna linjen, SW Horto AB, Sweden; NPK 14–16–18, pH 5.5–6.5), with two seedlings per pot. Plants were then propagated at 24 °C with 60% RH with a minimum light intensity of 300 μmol/m^2^ s for 16 h and a dark period for 8 h.

The experiment consisted of two treatments i.e., *Z. tritici* (Zt) alone and *Z. tritici* with *C. rosea* (ZtCr). In each treatment, three replicates were used. For treatment Zt, two replicates were evaluated in 2019 [39] and one replicate in 2022, whereas for ZtCr, all three replicates were evaluated in 2022. In each replicate, the pots were randomized in an augmented design using the R package agricolae [40]. Eight to nine blocks of test genotypes with four check genotypes were used in each replicate.

Foliar application of *C. rosea* and *Z. tritici* was done in the same way as in the preliminary small-scale experiment. Plants were sprayed until run-off with *C. rosea* suspension at the concentration of 1 × 10^7^ cfu/ml in the treatment ZtCr (and with water only in the treatment Zt) and were incubated at 90% RH. After 24 h, plants were sprayed until run-off with *Z. tritici* at the concentration of 1e10^6^ cfu/ml in both Zt and ZtCr treatments and were incubated at 90% relative humidity for 48 h. To maintain high humidity, plants were also sprinkled with water using a sprinkler hose four to five times per day. Disease was assessed on two fully developed leaves marked at the base using a marker pen before the inoculation. Percentage of leaf necrotic area was used as a proxy for disease and was visually scored from 0 to 100% with 5% step interval (Fig. 1A). Disease scoring was done at three time points (13, 16 and 19 dpi) in 2019 and at four time points (10, 13, 16 and 20 dpi) in 2022 and the disease progress over time was summarized by estimating rAUDPC.

## Phenotypic data analysis

### Small-scale biocontrol efficacy screening

To check for the genotypic differences between treatments, analysis of variance (ANOVA) was performed using a linear mixed model with genotype and treatment interaction. The model is as follows:

$${y}_{ijkl}=\mu +{g}_{i}+{t}_{l}+{(gt)}_{il}+{p}_{lj}+{n}_{ljk}+ {\varepsilon }_{il}$$

where ${y}_{ijkl}$ was the rAUDPC estimate of the *i*-th genotype in the *l*-th treatment, μ denotes the overall mean, ${g}_{i}$ is the effect of the *i*-th genotype, ${t}_{l}$ is the effect of *l*-th treatment, ${(gt)}_{il}$ is the interaction effect of the *i*-th genotype with the *l*-th treatment, ${p}_{lj}$ is the effect of the *j*-th pot nested in the *l*-th treatment, ${n}_{ljk}$ is the effect of the *n*-th plant nested within the *j*-th pot in the *l*-th treatment, and ${\varepsilon }_{il}$ is the residual term for which homogenous variance was assumed and was subjected to normal distribution. Pots and plants within pots were considered as random factors in the model. In addition, for multiple comparisons, a post-hoc Tukey’s test among genotypes across treatments was performed.

### Large-scale biocontrol efficacy screening

Disease scores within a pot (replicate) were averaged at each time point prior to rAUDPC estimation. Within each replicate, rAUDPC values were centered and scaled to account for scoring on different days. Therefore, the mean estimate at each replicate level, and ultimately, also at treatment level was centered to 0. However, the absolute rankings of genotypes and the difference among genotypes was still maintained to study genotype level differences. The linear mixed model analysis using Kenward-Roger's approximation of the degrees of freedom [41] to estimate best linear unbiased estimators (BLUEs) was performed on these centered and scaled rAUDPC values in the following way:

#### Intra treatment

ANOVA was performed for all rAUDPC separately in each treatment using the following linear mixed model:

$${y}_{ijk}=\mu +{g}_{i}+{r}_{j}+{b}_{jk}+{\varepsilon }_{ijk}$$

where ${y}_{ijk}$ was the phenotypic performance of the *i*-th genotype in the *k*-th block nested within the *j*-th replicate, μ denotes the overall mean, ${g}_{i}$ is the effect of the *i*-th genotype, ${r}_{j}$ is the effect of the *j*-th replicate, ${b}_{jk}$ is the effect of the *k*-th block nested within the *j*-th replicate, and ${\varepsilon }_{ijk}$ is the residual term. Blocks nested within replicates were treated as a random factor. Broad-sense heritability was also estimated in treatments Zt and ZtCr as H^2^_P_ after Piepho & Möhring [42] and H^2^_C_ after Cullis et al*.* [43] as below:

$${H}_{P}^{2}= \frac{{\sigma }_{g}^{2}}{{\sigma }_{g}^{2}+ {\overline{\nu }}_{BLUE}/2}$$

where ${\sigma }_{g}^{2}$ is the genotypic variance and ${\overline{\nu }}_{BLUE}$ is the mean variance of a difference of two genotypic BLUEs

$${H}_{C}^{2}=1- \frac{{\overline{\nu }}_{BLUP}}{2{\sigma }_{g}^{2}}$$

where ${\overline{\nu }}_{BLUP}$ is the mean variance of a difference of two best linear unbiased predictors (BLUPs) for the genotypic effect and ${\sigma }_{g}^{2}$ is the genotypic variance.

#### Inter treatment

To check for the genotypic differences between treatments, a full mixed model with genotype and treatment interaction was applied. Genotypes that were not present in both treatments were removed before the analysis (183 genotypes overlapping between treatments). The model is as follows:

$${y}_{ijkl}=\mu +{g}_{i}+{t}_{l}+{(gt)}_{il}+ {r}_{lj}+ {b}_{ljk}+ {\varepsilon }_{ijkl}$$

where ${y}_{ijkl}$ was the phenotypic performance of the *i*-th genotype in the *k*-th block nested within the *j*-th replicate in the *l*-th treatment, μ denotes the overall mean; ${g}_{i}$ is the effect of the *i*-th genotype, ${t}_{l}$ is the effect of *l*-th treatment, ${(gt)}_{il}$ is the interaction effect of of the i-th genotype with the *l*-th treatment, ${r}_{lj}$ is the effect of the *j*-th replicate nested in the *l*-th treatment, ${b}_{ljk}$ is the effect of the *k*-th block nested within the *j*-th replicate in the *l*-th treatment, and ${\varepsilon }_{ijkl}$ is the residual term. Blocks nested within replicates in treatments were treated as a random factor. To estimate differences between treatments for each genotype, a post-hoc Tukey’s test was also performed. Specifically, the contrasts between treatments, calculated as the difference between Zt (disease severity with *Z. tritici* alone) and ZtCr (disease severity with both *Z. tritici* and *C. rosea*), were used as measures of biocontrol efficacy (Zt—ZtCr). These contrasts served as indicators of the effect of *C. rosea* in reducing disease severity for each genotype.

All statistical analyses were performed using the statistical software R version 4.1.1 “Kick Things” [44]. The linear mixed model analysis was performed using lmer package [45] and its extension lmertest [46]. In addition, post-hoc comparisons among genotypes at treatment level and individual genotype comparison between treatments were performed using emmeans [47] and cld [48] R packages. Tidyverse suite [49] was used for most of the data processing and visualization alongside other dependency packages.

### Genome-wide association analysis

For GWA mapping, the Genome association and prediction integrated tool (GAPIT) in the R environment was used [50]. The wheat genotypes were previously genotyped using a 20 K SNP marker array [39]. SNP markers with > 20% missing alleles were removed. The remaining missing values were imputed using GAPIT to major alleles. For GWA analyses, a threshold of 5% minor allele frequency was applied. After quality checks, 7360 SNP markers were left for the GWAS (Supp. Table 2). In total, five different models were used: GLM [51], MLM [52], MLMM [53], FarmCPU [54] and BLINK [55]. GLM and MLM are single locus GWA models while MLMM, FarmCPU and BLINK are multiple loci GWA models. The kinship matrix (K) and the first ten principal components (PC) were used as covariates to adjust for familial relatedness and population structure. Only genotypes where SNP marker information was available were included in the GWA analyses. Genotypic marker data was available for 188 genotypes from treatment Zt and 173 individuals from treatment ZtCr and inter-treatment genotype contrasts for biocontrol efficacy. Alongside Bonferroni threshold (0.05/number of SNP markers) for marker-trait association, a less stringent threshold of negative log (1/number of SNP markers) was used to account for relatively low sample size and over stringency of Bonferroni test [56, 57]. For each SNP marker significant at negative log threshold, allelic level comparisons with one-way ANOVA were also made for associated traits.

### Identification of candidate genes

We used two methods to define regions in which to search for genes localized at significant marker trait associations. Firstly, as per linkage disequilibrium based criteria explained in Alemu et al. [58], a region of ± 1.6 cM flanking the significantly associated SNP marker was defined as a single quantitative trait locus (QTL) in this germplasm using the same SNP marker chip. The physical positions of SNP markers flanking the ± 1.6 cM region were identified by mapping SNP marker sequences against the *T. aestivum* IWGSC CS RefSeq v2.1 genome (GCF_018294505.1) using the BLAST algorithm with a threshold E-value of < 1E-30. Additionally, a more stringent criteria of ± 100 Kbp was also applied to define regions where the physical positions of flanking SNP markers were identified in the same manner and were used in gene annotation. Sequence matches with > 90% identity on the known chromosome locations of the SNPs were used to map them onto the *T. aestivum* IWGSC CS RefSeq v2.1 genome. The genes localized within the physical location of flanking SNP markers were filtered using the gene annotation data of *T. aestivum* (version 55) from the EnsemblPlants database. Only the protein coding gene models assigned to high confidence according to International Wheat Genome Sequencing Consortium (IWGSC) were used for annotation. The predicted amino acid sequences of the filtered genes were annotated by integrating annotation information obtained from various databases, using BLAST at the National Centre for Biotechnology Information (NCBI), and by Conserved Domain Search [59], the SMART analysis tool [60] and InterproScan [61]. Results from these databases were cross-referenced and summarized to assign putative functions to each gene based on annotations across tools.

## Results

### Biocontrol of septoria tritici blotch disease

To evaluate biocontrol of STB by *C. rosea*, a pilot experiment was performed using two susceptible (Nimbus and Kask) and two resistant (SW_150428 and Festival) genotypes. The experiment resulted in STB disease development over time (rAUDPC) in susceptible cultivars and a significant reduction in STB disease in treatments with *C. rosea* (Fig. 1B, Supp. Table 3). The ANOVA test on rAUDPC showed significant genotype-treatment interaction (*P* < 0.001), indicating differences among genotypes and treatments within genotypes, which were further explored using multiple comparisons Tukey test. No disease development in treatments control and Cr 1 × 10^8^ in any of the four genotypes (Fig. 1B, Supp. Table 3) was detected. In resistant genotypes SW_150428 and Festival, no significant disease development was observed in any of the treatments (Fig. 1B, Supp. Table 3). The highest rAUDPC value was observed in the treatment Zt 1 × 10^6^ for susceptible genotypes Nimbus (0.48) and Kask (0.21), which was significantly (*P* < 0.05) higher than the mock-inoculated controls (Fig. 1B, Supp. Table 3). These rAUDPC estimates in the treatment Zt 1 × 10^6^ were significantly (*P* < 0.05) higher than the three treatments (Cr 1 × 10^8^ & Zt 1 × 10^6^, Cr 1 × 10^8^ & Zt 5 × 10^5^ and Cr 1 × 10^8^ & Zt 1 × 10^5^) that involved *C. rosea* on the susceptible genotypes, exhibiting the biocontrol effect of *C. rosea* in controlling STB (Fig. 1B, Supp. Table 3).

### Variation among wheat genotypes for septoria tritici blotch disease 

**Fig. 2:**
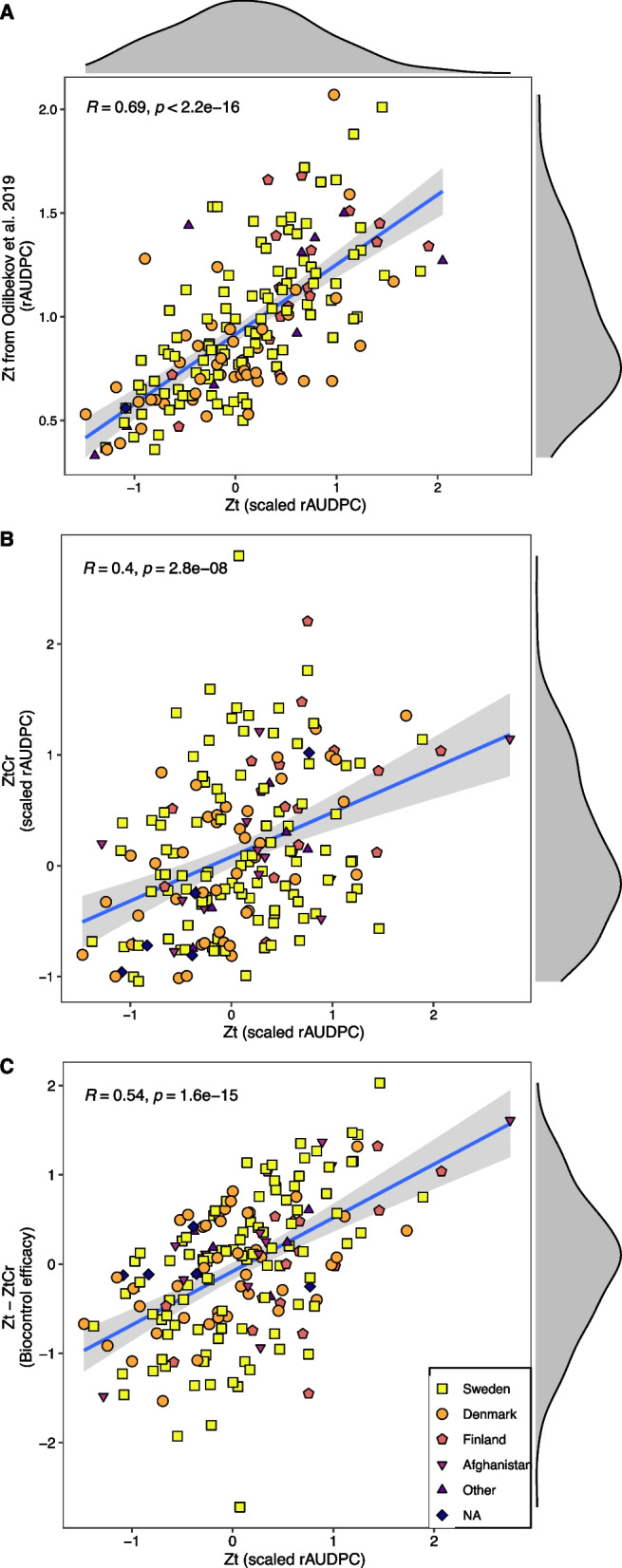
Correlations between treatments and traits. Pearson’s correlation for relative area under disease progress curve (rAUDPC) between different treatments and traits was calculated. (**A**) Correlation between scaled rAUDPC estimates in treatment Zt (*Z. tritici* only) against rAUDPC estimates in Odilbekov et al., (2019), (**B**) Correlation between scaled rAUDPC estimates in treatment Zt (*Z. tritici* only) against rAUDPC estimate in treatment ZtCr (*Z. tritici* along with *C. rosea*), and (**C**) Correlation between scaled rAUDPC in treatment Zt (*Z. tritici* only) against biocontrol efficacy estimates (Zt—ZtCr) of *C. rosea* in controlling septoria tritici blotch. Points with different shapes and color represent the country of origin of wheat genotypes. Plot margins show distribution of respective treatments

Application of *Z. tritici* alone (treatment Zt) to 202 winter wheat genotypes resulted in disease development with a varying degree among genotypes. Evaluation of rAUDPC in treatment Zt using the intra treatment linear mixed model showed significant variation (*P* < 0.001) for disease severity among genotypes (Table 1, Supp. Figure 1 A, Supp. Table 4). The rAUDPC in treatment Zt showed moderate to high heritability (H^2^_P_ = 0.67, H^2^_C_ = 0.59) and these results were in strong positive correlation (*R* = 0.69, *P* < 0.001) with STB rAUDPC data reported in Odilbekov et al*.* [39] where the same plant material was used for STB disease assessment (Table 1, Fig. 2A).

**Table 1:** Analysis of variance summary from linear mixed models for septoria tritici blotch disease resistance and biocontrol

Model^1^	Parameter	Sum of Squares	Mean Squares	NumDF^2^	DenDF^3^	F-value	P-value
***Intra treatment***							
Zt	Rep	3.4703	1.73515	2	17.13	63.1694	1.24E-08***
	Gen	15.0971	0.07511	201	401.14	2.7347	< 2.2E-16***
	H^2^_P_	0.67					
	H^2^_C_	0.59					
ZtCr	Rep	0.7074	0.3537	2	19.93	12.7986	0.0002662***
	Gen	15.3231	0.08419	182	433.97	3.0465	< 2.20E-16***
	H^2^_P_	0.74					
	H^2^_C_	0.62					
***Inter treatment***	Trt (Rep)	17.6382	0.09691	182	813.33	3.4507	< 2.20E-16***
	Gen	0.4304	0.43041	1	36.72	15.3263	0.0003775***
	Trt	7.3341	0.0403	182	813.33	1.4348	0.0005701***
	Gen × Trt	3.6963	0.92407	4	36.17	32.9048	1.35E-11***

^1^Models include intra treatment models fit separately in treatments Zt (disease with only *Z. tritici*), ZtCr (disease with *Z. tritici* in presence of biocontrol agent *C. rosea*). Inter treatment model includes Genotype × Treatment interaction

^2^Numerator degrees of freedom

^3^Denominator degrees of freedom

*Abbreviations:* *Rep* Replicate, *Gen* Genotype, *H*^*2*^_*P*_ Heritability (Piepho & Möhring, 2007), *H*_*2*_^*C*^ Heritability (Cullis et al*.*, 2006), *Trt* Treatment

Application of *C. rosea* formulation to the leaves before *Z. tritici* application (treatment ZtCr) also resulted in significant (*P* < 0.001) differences in disease severity between wheat genotypes with moderate to high heritability (H^2^_P_ = 0.74, H^2^_C_ = 0.62) (Table 1, Supp. Figure 1B, Supp. Table 5). There was a moderate positive correlation (*R* = 0.4, *P* < 0.001) between the treatments Zt and ZtCr (Fig. 2B). This moderate positive correlation reflects the changes in disease development in wheat genotypes in presence of *C. rosea*.

### Variation among wheat genotypes for *C. rosea* biocontrol efficacy

To quantify differences in rAUDPC values for each genotype between the treatments with *Z. tritici* alone (Zt) and *Z. tritici* with BCA *C. rosea* (ZtCr), the inter treatment linear mixed model with treatment and genotype interaction effect was employed. A total of 183 genotypes were overlapping between the treatments and were used in the analysis. Identical to the within-treatment analysis in the previous section, significant (*P* < 0.001) variation was observed for STB disease development estimates (rAUDPC) among genotypes in the treatments Zt and ZtCr. The model also showed significant interaction (*P* < 0.001) between genotype and treatment effect indicating differences in rAUDPC values for genotypes between treatments Zt and ZtCr (Table 1).

**Fig. 3:**
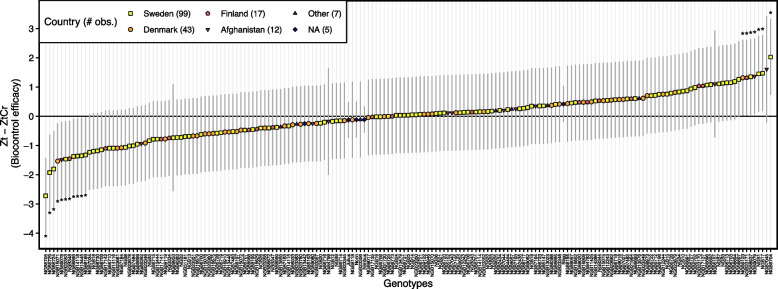
Biocontrol of septoria tritici blotch by *Clonostachys rosea*. Biocontrol efficacy estimates (Zt—ZtCr) of *C. rosea* in controlling septoria tritici blotch for 183 winter wheat genotypes was calculated. Scaled relative area under disease progress curve (rAUDPC) was estimated in treatments Zt (*Z. tritici* alone) and ZtCr (*Z. tritici* along with *C. rosea*) for each wheat genotype using linear mixed models. Post-hoc comparisons using Tukey’s test were used to estimate differences between treatment Zt and ZtCr for each genotype and were used as estimators for biocontrol efficacy. Points represent the model estimated means and error bars represent 95% confidence intervals for each genotype. Genotypes with ‘*’ (*P* < 0.05) highlight significant difference in rAUDPC estimates between treatments Zt and ZtCr and therefore, represent significant biocontrol efficacy. Points with different shapes and color represent the country of origin of wheat genotypes

The rAUDPC differences between treatments (Zt—ZtCr) for each genotype were used as an estimator for biocontrol efficacy and were estimated using Tukey’s multiple comparison test. Post-hoc comparison of 183 wheat genotypes for rAUDPC between Zt and ZtCr revealed a varying degree of disease difference between treatments (i.e. biocontrol efficacy) ranging from more disease in Zt for some genotypes to more disease in ZtCr (negative values) for other genotypes (Fig. 3, Supp. Table 6). In particular, seven genotypes (NGB9123, NGB2317, NGB8937, NGB9079, NGB1, NGB17 and NGB6704) had a significant (*P* < 0.05) positive effect of *C. rosea* biocontrol efficacy as they showed higher rAUDPC estimates in treatment Zt than ZtCr (Fig. 3, Supp. Table 6). On the other hand, eleven genotypes (NGB6705, NGB6729, NGB6724, NGB15071, NGB9078, NGB23353, NGB348, NGB6699, NGB14118, NGB13445 and NGB8189) had a significant (*P* < 0.05) negative effect of *C. rosea* with higher rAUDPC estimates in treatment ZtCr than Zt (Fig. 3, Supp. Table 6). *Clonostachys rosea* biocontrol efficacy estimates were also found to be in significant moderate positive correlation (*R* = 0.54, *P* < 0.001) with rAUDPC estimates from treatment Zt, suggesting that susceptible genotypes benefit more from *C. rosea* application as more reduction in disease was observed (Fig. 2C).

### Genome-wide marker trait associations

For phenotypic rAUDPC estimates in treatments with *Z. tritici* alone (Zt), *Z. tritici* along with BCA *C. rosea* (ZtCr) and biocontrol efficacy estimator (Zt—ZtCr), Genome-wide association analysis was performed using 20 K SNP marker array genotyping data with 7360 markers. The GWAS detected eleven SNP markers (eight of these SNP markers with multi-model GWAS) that were significantly (*P* ≤ 0.00014, after *P* ≤ 1/n, where n = 7360 is the number of SNP markers) associated to rAUDPC variation (Table 2). Seven out of these eleven SNP marker-trait associations were co-detected by more than one GWAS model (Table 2).

**Table 2:** Summary of GWAS results for significant SNP markers associated with Zt (*Z. tritici* alone), ZtCr (*Z. tritici* along with *C. rosea*) and biocontrol efficacy (Zt—ZtCr) estimates in winter wheat genotypes

Trait	SNP marker	Chromosome	Physical position (bp)	Genetic position (cM)	maf	PVE (%)	Model	P.value	Allele effect
Zt (*Z. tritici* alone)	Excalibur_c49875_479	2B	788,524,048	145	0.19	4.0	Blink	6.55E-08	NA
							FarmCPU	4.60E-05	−0.36
							MLMM	6.17E-05	NA
	IAAV4876	3B	3,492,598	51	0.09	6.6	GLM	1.31E-04	−0.39
	Kukri_rep_c70198_1436	3B	495,024,661	68	0.13	5.1	Blink	4.72E-07	NA
	Excalibur_c29625_222	3B	493,110,618	68	0.14	7.6	GLM	4.10E-05	−0.32
	RAC875_rep_c83245_239	3B	493,776,130	68	0.15	7.2	GLM	6.14E-05	−0.31
ZtCr (*Z. tritici* along with *C. rosea*)	BS00022902_51	1B	73,310,732	59	0.33	3.8	Blink	8.53E-05	NA
							FarmCPU	8.53E-05	−0.29
							MLMM	9.95E-05	NA
	BS00070856_51	6D	494,584,503	153	0.20	0.7	Blink	1.05E-04	NA
							FarmCPU	1.05E-04	0.36
Zt – ZtCr (Biocontrol efficacy)	Kukri_c837_436	1D	9,020,946	23	0.06	1.7	Blink	7.74E-06	NA
							FarmCPU	7.74E-06	0.75
							MLMM	1.32E-05	NA
	wsnp_Ex_c1358_2600929	1D	9,016,388	23	0.06	1.8	Blink	8.19E-05	NA
							FarmCPU	8.19E-05	0.63
	wsnp_Ex_c1358_2602235	1D	9,017,694	24	0.06	1.8	Blink	8.19E-05	NA
							FarmCPU	8.19E-05	0.63
	BS00027770_51	6B	700,313,370	98	0.23	1.7	Blink	2.68E-05	NA
							FarmCPU	2.68E-05	−0.41

*Abbreviations*: *GWAS* Genome-wide association study, *SNP* Single Nucleotide Polymorphism, *cM* centi-Morgan, *maf* minor allele frequency, *PVE* Percent Variation Explained

PVE (%) calculated as the percent difference between the R^2^ of the GLM model with and without the associated SNP

Allele effect estimates the additive contribution of the tested marker SNP marker

**Fig. 4:**
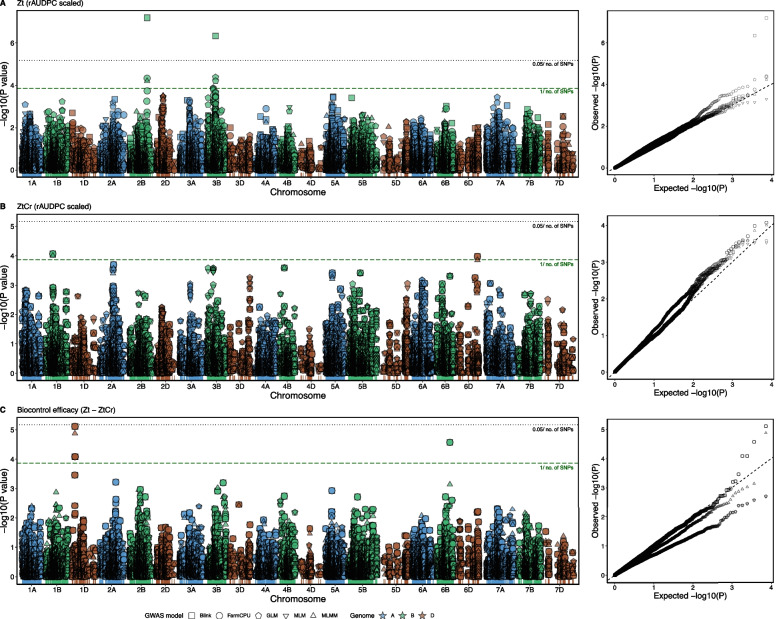
Manhattan plot (left) and Q-Q plot (right) for marker-trait association between for scaled relative area under disease progress curve (rAUDPC) of wheat genotypes in (**A**) treatment Zt (*Z. tritici* alone), (**B**) treatment ZtCr (*Z. tritici* along with *C. rosea*) and (**C**) Biocontrol efficacy (Zt – ZtCr) and 7360 single nucleotide polymorphism (SNP) markers from the genome–wide association study (GWAS) models. Black dotted line depicts the Bonferroni significance threshold (*P* = 0.05/n, where n = 7360 is the number of SNP markers), green dashed line depicts less stringent threshold (*P* = 0.00014, after *P* = 1/n, where n = 7360 is the number of SNP markers)

For the rAUDPC in treatment Zt, five SNP markers at three locations i.e., Excalibur_c49875_479 (chromosome 2B with BLINK, FarmCPU and MLMM model), IAAV4876 (chromosome 3B at 51 cM with GLM model), Excalibur_c29625_222 (chromosome 3B at 68 cM with GLM model), Kukri_rep_c70198_1436 (chromosome 3B at 68 cM with BLINK model) and RAC875_rep_c83245_239 (chromosome 3B at 68 cM with GLM model), were found to be significantly associated (*P* ≤ 0.00014) (Table 2, Fig. 4A). Percent variation explained by these markers ranged from 4% to 7.6% (Table 2). At allelic level, genotypes that carried the AA allele for SNP marker IAAV4876 exhibited significantly (*P* < 0.05) lower rAUDPC value (Supp. Figure 2B).

For treatment ZtCr in presence of BCA *C. rosea*, two SNP markers i.e., BS00022902_51 (chromosome 1B with BLINK, FarmCPU and MLMM model) and BS00070856_51 (chromosome 6D with BLINK and FarmCPU model), were significantly (*P* ≤ 0.00014) associated with rAUDPC estimates (Table 2, Fig. 4B). At allelic level, genotypes that carried the allele TT for SNP marker BS00070856_51 exhibited significantly (*P* < 0.05) lower rAUDPC value (Supp. Figure 2G).

For biocontrol efficacy (Zt—ZtCr), significant (*P* ≤ 0.00014) SNP marker-trait associations were detected at two locations i.e., at chromosome 1D by SNP markers Kukri_c837_436 (BLINK, FarmCPU and MLMM model), wsnp_Ex_c1358_2600929 (BLINK and FarmCPU) and wsnp_Ex_c1358_2602235 (BLINK and FarmCPU), and at chromosome 6B by SNP marker BS00027770_51 (BLINK and FarmCPU) (Table 2, Fig. 4C). Percent variation explained by these markers was 1.7% to 1.8% (Table 2). At allelic level, genotypes that carried the alleles GG and GT for SNP marker Kukri_c837_436, alleles AA and AG for SNP marker wsnp_Ex_c1358_2600929 and alleles CC and CT for SNP marker wsnp_Ex_c1358_2602235 showed significantly (*P* < 0.05) more biocontrol efficacy (Supp. Figure 2H-J). Moreover, genotypes with allele AA for SNP marker BS00027770_51 showed significantly (*P* < 0.05) more biocontrol efficacy (Supp. Figure 2K).

### Gene content in genomic regions with associated SNP markers

The regions around seven locations (three locations for treatment Zt, two locations for treatment ZtCr and two locations for biocontrol efficacy) with significant marker-trait associations were defined using linkage disequilibrium-based criteria defined in Alemu et al*.* [58]. Moreover, a less stringent criteria of ± 100 Kbp flanking the significant markers were used to explore genes. In regions using the criteria of ± 1.6 cM flanking the significant SNP markers, the regions spanned from 0.7 Mbp (for biocontrol efficacy at 1D) to 88.4 Mbp (treatment Zt at chromosome 3B at 68 cM). In total, the number of genes in these regions with assigned high confidence according to IWGSC were 1290 for treatment Zt, 92 for treatment ZtCr and 61 for biocontrol efficacy (Zt – ZtCr) (Supp. Table 7).

Using the more stringent criteria of ± 100 Kbp to define regions, twenty genes were found to be localized within the genomic regions surrounding the physical position of GWAS-identified SNP markers significantly (*P* ≤ 0.00014) associated with treatments Zt, ZtCr and biocontrol efficacy (Zt – ZtCr) (Table 3). Fifteen genes were localized within ± 100 Kbp intervals of SNP markers associated with treatment Zt for disease severity (Table 3). TraesCS3B02G307000 was predicted to encode a protein with sequence similarity to a plant homeodomain (PHD) Zinc finger-type pathogenesis-related transcription factor in *Arabidopsis thaliana*. Other predicted proteins with putative functions in plant defense and stress mitigation included a NUDIX domain-containing protein, a cytochrome P450 monooxygenase, a dynamin-like GTPase, VQ motif-containing protein and a carotenoid cleavage dioxygenase (Table 3).

**Table 3:** Gene content of wheat genomic regions segregating with septoria tritici blotch disease resistance and biocontrol in ± 100 Kbp interval

Trait	Chromosome	Physical position (bp)	Gene	Putative function
Disease resistance (Zt)	2B	788,424,048—788,624,048	TraesCS2B02G608100	NUDIX domain-containing protein
			TraesCS2B02G608200	Extracellular protein with unknown function
	3B	3,392,598—3,592,598	TraesCS3B02G006500	60S ribosomal protein L6
			TraesCS3B02G006600	Cytochrome P450 family 86
	3B	493,010,618—495,124,661	TraesCS3B02G307000	Zinc finger pathogenesis-related transcription factor
			TraesCS3B02G307100	Dynamin-like GTPase
			TraesCS3B02G307200	Uncharacterized protein
			TraesCS3B02G307300	VQ motif-containing protein
			TraesCS3B02G307400	RNA binding domain-containing protein
			TraesCS3B02G307500	Pentatricopeptide repeat-containing protein
			TraesCS3B02G307600	Dihydroorotase enzyme for pyrimidine biosynthesis
			TraesCS3B02G307700	A-Raf-like serine/threonine-protein kinase
			TraesCS3B02G307800	Uncharacterized protein
			TraesCS3B02G307900	Mannan endo-1,4-beta-mannosidase 5 like protein
			TraesCS3B02G308000	Carotenoid cleavage dioxygenase
Biocontrol (ZtCr)	1B	73,210,732—73,410,732	-	
	6D	494,484,503—494,684,503	-	
Biocontrol efficacy (Zt—ZtCr)	1D	8,920,946—9,120,946	TraesCS1D02G020800	Mechanosensitive channel protein
			TraesCS1D02G020900	Myb-like transcriptional regulator
			TraesCS1D02G021000	Pik-2-like disease resistance protein
			TraesCS1D02G021100	AKR4 C-type aldo–keto reductase
			TraesCS1D02G021200	Pik-2-like disease resistance protein
	6B	700,213,370—700,413,370	-	

Putative function assigned after cross-referencing NCBI, Conserved Domain Search, SMART and InterproScan databases

No genes were identified in the genomic regions segregating with biocontrol treatment ZtCr (Table 3). However, five genes (TraesCS1D02G020800, TraesCS1D02G020900, TraesCS1D02G021000, TraesCS1D02G021100 and TraesCS1D02G021200) were localized within intervals of SNP markers associated with biocontrol efficacy Zt—ZtCr (Table 3). TraesCS1D02G021000 and TraesCS1D02G021200 were predicted to encode proteins with sequence similarity to Pik-2-like disease resistance proteins (Table 3). Other proteins present in the region were predicted to be involved in transcriptional regulation, mechanosensitive ion channels and oxidoreductase activities (Table 3).

## Discussion

Utilization of genetic variability through breeding is one of the main ways to improve yield, quality, disease resistance and abiotic stress tolerance in agricultural plants. Likewise, novel traits of interest such as interactions with beneficial microorganisms, interactions with other plants and microbiome modulation can also benefit from a more in-depth understanding of genetic variation and genotype-genotype interactions [31, 62–64]. In this study, we explored the natural variation present in 202 winter wheat genotypes for STB disease resistance and variation in wheat genotypes affecting the biocontrol efficacy of *C. rosea* in controlling STB.

Using a large and diverse panel of winter wheat genotypes primarily from the Scandinavian countries, we found significant variation among wheat genotypes for both disease resistance and biocontrol efficacy of *C. rosea*. This panel of winter wheat genotypes has previously been used to explore genetic variation for several diseases such as powdery mildew, fusarium head blight, fusarium foot rot and STB [33, 39, 58, 65]. Our results for STB resistance are in agreement with data reported in Odilbekov et al*.* [39], where leaf necrosis was also used as a proxy for disease development. While pycnidia coverage is recognized as one of the main estimators of STB development and *Z. tritici* fitness [38, 66], we observed inconsistent pycnidia development and therefore focused solely on necrosis for our analysis.

*Clonostachys rosea* is primarily considered as a soil-borne and rhizosphere-associated fungus that has shown biocontrol properties against a multitude of diseases [10]. However, *C. rosea* is reported to act as a BCA against several diseases in the phyllosphere caused by pathogens such as *F. graminearum*, *Puccinia triticana*, *P. hordei*, *P. coronata* f. sp. *avenacea* and *B. sorokiniana* [10, 15, 16, 67]. In this study, we have shown that application of *C. rosea* strain IK726 to wheat leaves efficiently protects against STB disease caused by *Z. tritici* in certain wheat genotypes under controlled conditions, while other genotypes showed no benefit or even a negative effect from the BCA treatment. However, without a *C. rosea* only control, it is not possible to determine if *C. rosea* contributed to the observed necrosis symptoms*.* Future studies should further investigate the direct effects of *C. rosea* on plant genotypes. Biocontrol of STB by *C. rosea* strain IK726 is previously reported from multi-year field trials in Denmark, where *C. rosea* IK726 alone and in combination with other BCAs showed significant reduction in STB compared to untreated control [17].

The importance of plant genotypes in BCA establishment, efficient biocontrol and biostimulation from beneficial microorganisms has been suggested before [26, 62]. However, knowledge about the extent and strength of this phenomenon is limited as only a small number of studies, with low numbers of plant genotypes tested, confirm plant host genotype-specific interactions with BCAs and biostimulants [27–30]. Here, we have used 183 wheat genotypes to show that the efficacy of *C. rosea* strain IK726 in controlling STB is quantitatively modulated by the plant genotype. The moderate positive correlation between disease resistance and biocontrol efficacy shows that susceptible plant genotypes typically benefit from *C. rosea* application. However, the fact that this correlation is not strong shows that there is an additional level of genetic predisposition in the wheat material to benefit from the BCA treatment. To gain deeper insights into the dynamics of the interaction between *Z. tritici* and *C. rosea*, future studies could focus on quantifying both the pathogen and the BCA on wheat leaves at various time points post-inoculation.

Large scale genetic studies in wheat have found several quantitative trait loci (QTLs) throughout the wheat genome associated with STB disease resistance [66, 68, 69]. Odilbekov et al*.* [39] found QTLs associated with STB on chromosomes 1 A, 1B, 2B, 3 A and 5 A. In this study, we identified two significant marker trait associations on chromosome 2B and 3B, which has also been reported previously on these chromosomes [39, 58, 66, 68, 70]. Various QTLs on these chromosomes contain known Stb genes which are suggested to contribute to disease resistance specifically at seedling stage and at adult plant stage, reflecting the complexity and variety of putative resistance genes linked to STB [39, 66, 68]. Thauvin et al*.* [70] identified a QTL on chromosome 2B (790,454,171–808,459,904 bp) that colocalizes with the *Stb9* major gene, near the QTL identified in our study. Additionally, Kumar et al*.* [71] identified a QTL associated with STB on chromosome 2B, located at 784,545,663 ± 2,436,999 bp, which is in close proximity to the regions we identified. Furthermore, Riaz et al*.* [72] reported QTLs for STB infection under field conditions on chromosomes 2B and 3B, with overlapping physical positions. In these regions, several genes predicted to have a role in plant defenses were also localized. The TraesCS3B02G307000 gene is predicted to encode for plant homeodomain (PHD) Zinc finger-type protein known to transcriptionally regulate plant defense genes, specifically pathogenesis related protein 2 [73]. Pathogenesis-related protein 2 exhibits β−1,3 glucanase enzymatic activity and plays a role in the hydrolysis of microbial cell walls [74, 75]. TraesCS3B02G307300 is a gene predicted to encode for a VQ motif-containing protein, which are known to regulate various developmental process including responses to biotic and abiotic stress generally in all plants [76] and in wheat [77]. TraesCS3B02G307100 is a predicted dynamin-like GTPase similar to members of dynamin superfamily that are involved in budding of transport vesicles, division of organelles, cytokinesis and pathogen resistance by mediating vesicle trafficking [78]. The presence of these resistance sources in Nordic wheat germplasm reflects a genetic potential that can be utilized in breeding to improve STB resistance in wheat, however, plant genotype specific variation in *C. rosea* efficacy under field conditions should be further evaluated.

We further identify significant associations of SNP markers to *C. rosea* biocontrol on chromosome 1B and 6D and biocontrol efficacy on chromosomes 1D and 6B, which are distinctive from marker-trait associations found for disease resistance. The phenotypic variation explained by significant markers for biocontrol efficacy is low, which may be due to low statistical power and/or due to the complexity of the trait. Nevertheless, these results indicate that the QTLs contributing to STB disease resistance and biocontrol are located in different genomic regions, which suggest that it is possible to breed wheat genotypes that combine high STB disease resistance with high BCA compatibility. SNP markers that exhibit segregation with distinct disease resistance and biocontrol related traits can aid in plant breeding by enabling the simultaneous and more efficient selection of multiple QTLs, offering a cost-effective approach. The underlying mechanisms for this plant genotype-dependent effect on biocontrol efficacy are currently not well understood. We identified two genes (TraesCS1D02G021000 and TraesCS1D02G021200) predicted to encode Pik-2 like disease resistance proteins, which were first shown to be R proteins inducing hypersensitive response in plants to restrict pathogen growth [79]. We also identified a paralog of Pik-2-like disease resistance protein segregating with the *C. rosea* mediated biocontrol efficacy of fusarium foot rot in the same winter wheat panel [33]. The presence of Pik-2-like disease resistance protein genes in regions segregating with biocontrol efficacy may suggest differential ability of wheat genotypes to recognize microbe-associated molecular patterns (MAMPs) or microbial effectors and subsequently in their ability to induce pattern-triggered immunity (PTI) or effector-triggered immunity (ETI) [10, 63, 80]. This is in line with results from Moraga-Suazo et al. [27] where *C. rosea*-mediated biocontrol of the pitch canker pathogen *F. circinatum* differs between *P. radiata* genotypes, which in turn is related with the ability of *C. rosea* to activate ISR. In fact, *C. rosea* can trigger defense gene expression in both wheat and tomato [21, 22, 81], which subsequently may trigger ISR as shown in wheat [82] and tobacco [83]. We further identified a gene (TraesCS1D02G020800) involved in mechanosensitive channel protein that provide protection against hypoosmotic shock [84, 85] and a gene (TraesCS1D02G020900) associated with a Myb-like protein involved in transcriptional regulation by DNA binding [86–88]. It is also possible that different wheat genotypes trigger varying levels of specialized metabolite production with antibiotic properties in *C. rosea* [89]. The fact that certain wheat genotypes responded negatively to the application of *C. rosea* in the presence of *Z. tritici* illustrates the delicate balance between BCAs, pathogens and plants at cellular and physiological level [10]. A potential mechanism could also involve *C. rosea* suppressing the PTI response in certain wheat genotypes, making them more susceptible to pathogen infection, although this hypothesis requires further investigation.

## Conclusions

This study highlights the role of plant genotypes in efficient application of BCAs. We showed that winter wheat genotypes vary in their ability to benefit from *C. rosea*-mediated biocontrol of STB and that disease resistance and biocontrol efficacy are genetically distinct traits. Breeding plants with a high genetic potential to benefit from the application of beneficial microorganisms can facilitate the transition to agricultural production systems with lower input of chemical fungicides. However, as we have emphasized previously [33], plant disease resistance must serve as the primary line of defense in an integrated disease management strategy. Therefore, any further advancements in selection for novel traits, such as breeding for compatibility with BCAs, should not undermine the existing disease resistance. Moreover, this study used single strains of *Z. tritici* and *C. rosea*, future research should explore the impact of diverse pathogen and BCA strains to further broaden these findings. Future studies to elucidate the underlying mechanisms of genotype-specific biocontrol efficacy, through approaches such as quantification of the pathogen and the BCA during infection, testing efficacy in field trials, and identifying and validating the involved genes, can further enhance the integration of beneficial microorganisms in sustainable agriculture.

## Supplementary Information

Supplementary Material 1. Supp. Figure 1: Phenotypic distribution of scaled relative area under disease progress curve (rAUDPC) of wheat genotypes in (A) treatment Zt (*Z. tritici* alone) and (B) treatment ZtCr (*Z. tritici* along with *C. rosea*). Points represent the model estimated means and error bars represent 95 % confidence intervals for each genotype. Points with different shapes and color represent the country of origin of wheat genotypes.

Supplementary Material 2. Supp. Figure 2: Allelic level comparison of SNP markers significantly associated with scaled relative area under disease progress curve (rAUDPC) of wheat genotypes in treatment Zt (*Z. tritici* alone), treatment ZtCr (*Z. tritici* along with *C. rosea*) and biocontrol efficacy (Zt – ZtCr). Panel A-F display significant markers with their location, their alleles and distribution of wheat genotypes for the associated trait. Alleles not sharing the same letter are significantly different at *P* < 0.05. Numbers at the bottom of the panel indicate number of genotypes, and black diamonds represent mean estimate of the group. 

Supplementary Material 3. Supp. Table 1: List of 202 winter wheat genotypes from NordGen gene bank with information on country of origin, year of origin, pedigree and inclusion in GWAS analysis.

Supplementary Material 4. Supp. Table 2: Single nucleotide polymorphism (SNP) marker information for 188 winter wheat genotypes used in the GWAS analyses.

Supplementary Material 5. Supp. Table 3: Small scale biocontrol efficacy screening experiment model output across treatments and genotypes.

Supplementary Material 6. Supp. Table 4: Model output for the treatment Zt (*Z. tritici* only) of 202 winter wheat genotypes.

Supplementary Material 7. Supp. Table 5: Model output for the treatment ZtCr (*Z. tritici* along with *C. rosea*) of 183 winter wheat genotypes.

Supplementary Material 8. Supp. Table 6: Biocontrol efficacy estimates (Zt - ZtCr) of *C. rosea* in controlling septoria tritici blotch for 183 winter wheat genotypes.

Supplementary Material 9. Supp. Table 7: Gene content of wheat genomic regions segregating with septoria tritici blotch disease resistance and biocontrol in ± 1.6 cM interval as per Alemu et. al. (2021).

## Data Availability

All the data produced in this study are available in supplementary files.

## Abbreviations

rAUDPC: Relative Area under disease progress curve
BCA: Biological control agent
GWAS: Genome–wide association study
IPM: Integrated pest management
QTL: Quantitative trait loci
SNP: Single nucleotide polymorphism
STB: Septoria tritici blotch
