# Supplementary figures and images for: Plant genotype-specific modulation of Clonostachys rosea-mediated biocontrol of septoria tritici blotch disease in wheat
Source: BMC Plant Biol. 2025 May 2;25:576. doi: 10.1186/s12870-025-06620-9 (PMC12049020; doi:10.1186/s12870-025-06620-9)

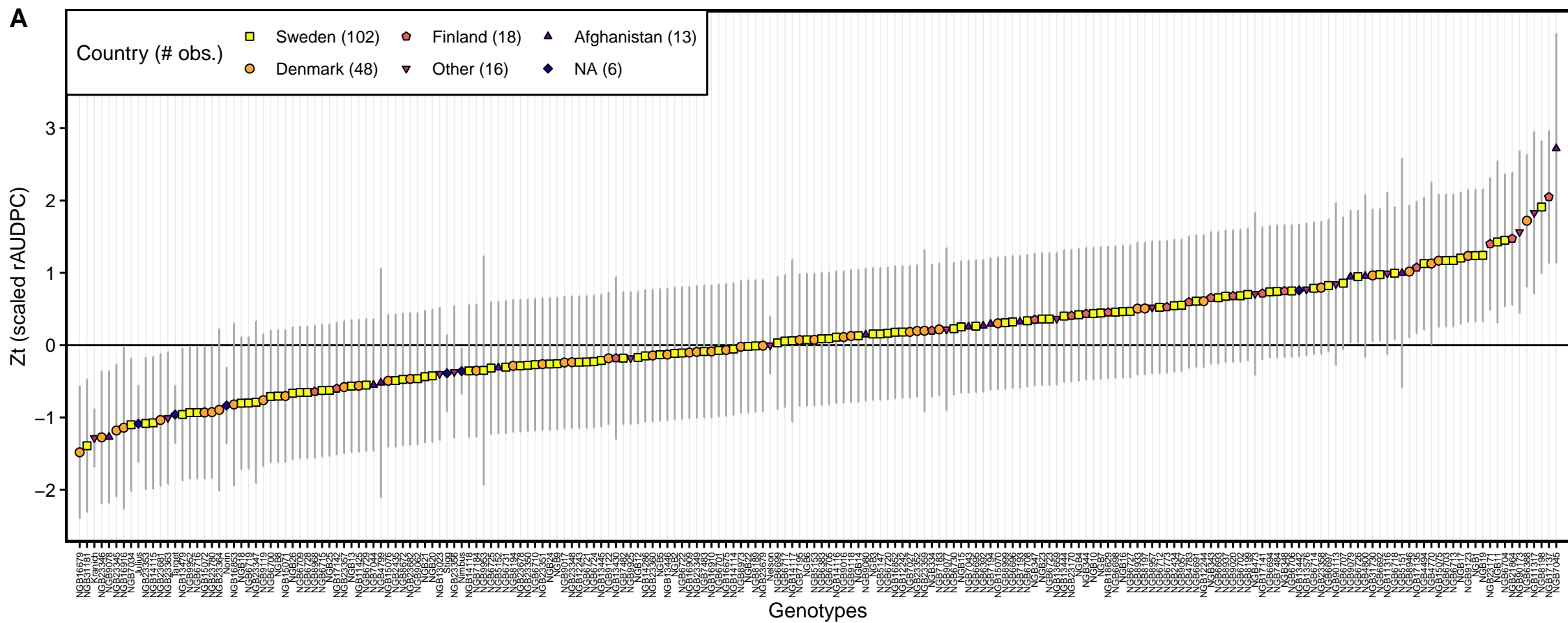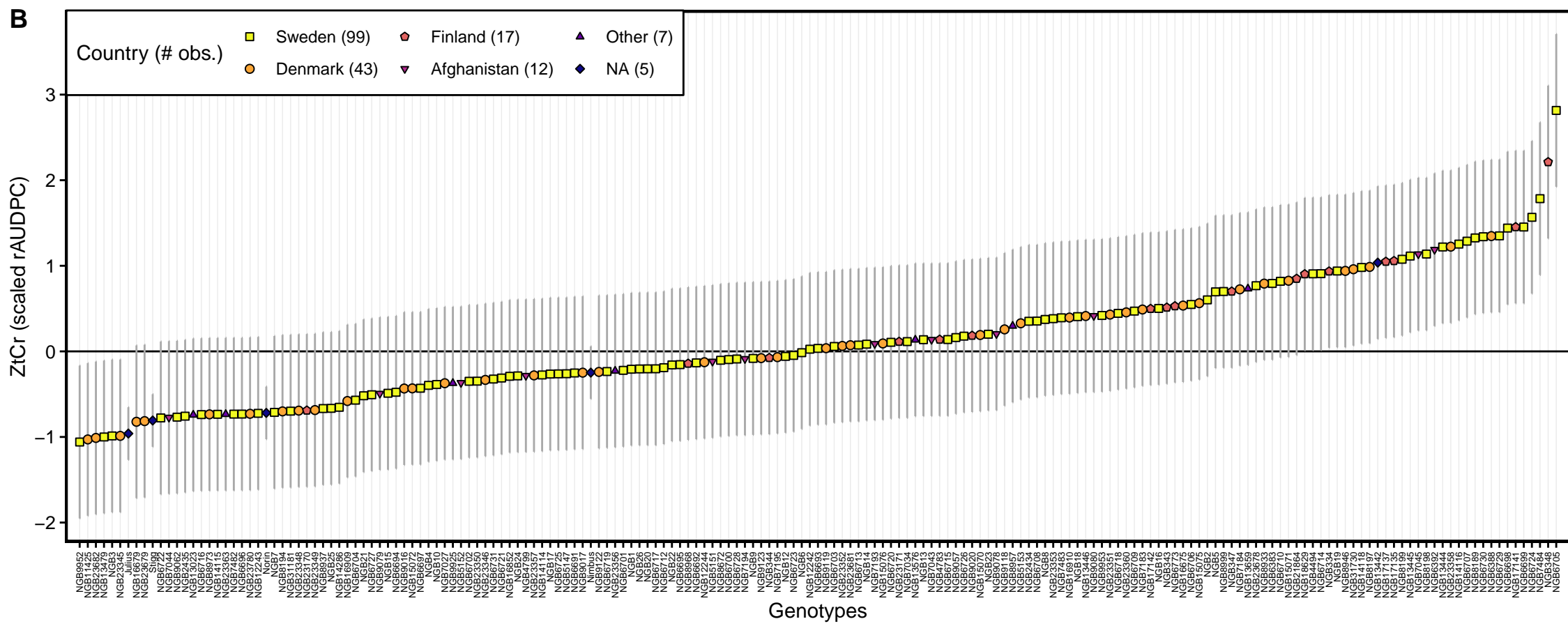

Supplement: Supplementary file 1 — Supplementary Material 1. Supp. Figure 1: Phenotypic distribution of scaled relative area under disease progress curve (rAUDPC) of wheat genotypes in (A) treatment Zt (Z. tritici alone) and (B) treatment ZtCr (Z. tritici along with C. rosea). Points represent the model estimated means and error bars represent 95 % confidence intervals for each genotype. Points with different shapes and color represent the country of origin of wheat genotypes. [file 12870_2025_6620_MOESM1_ESM.pdf]

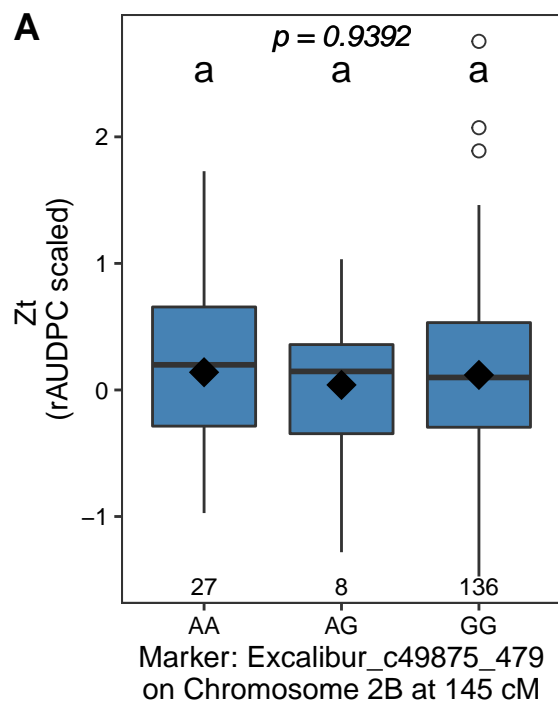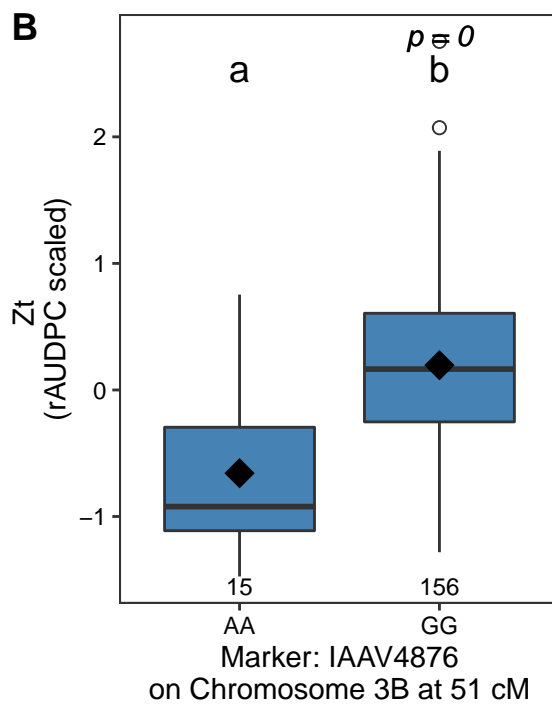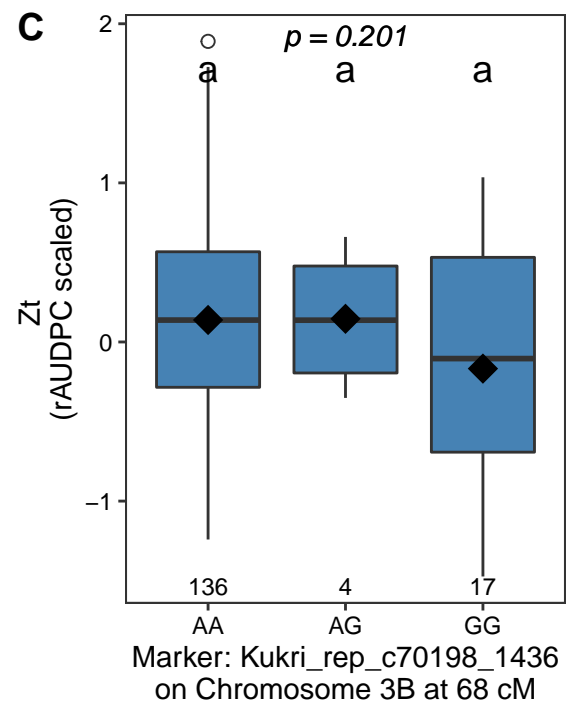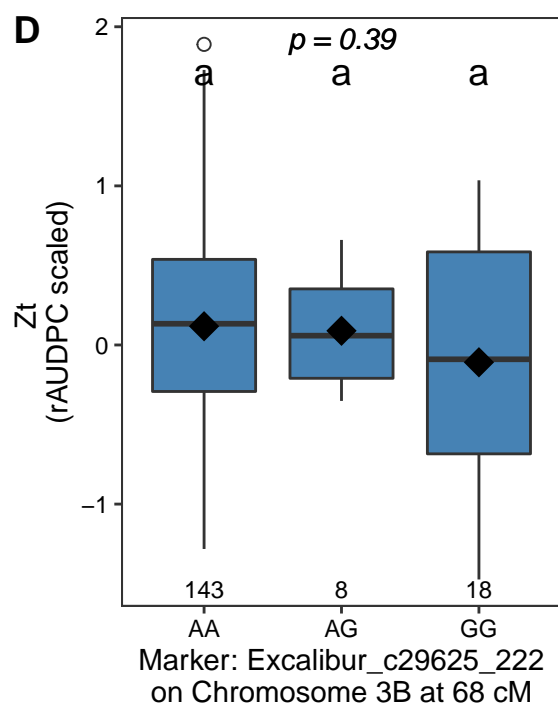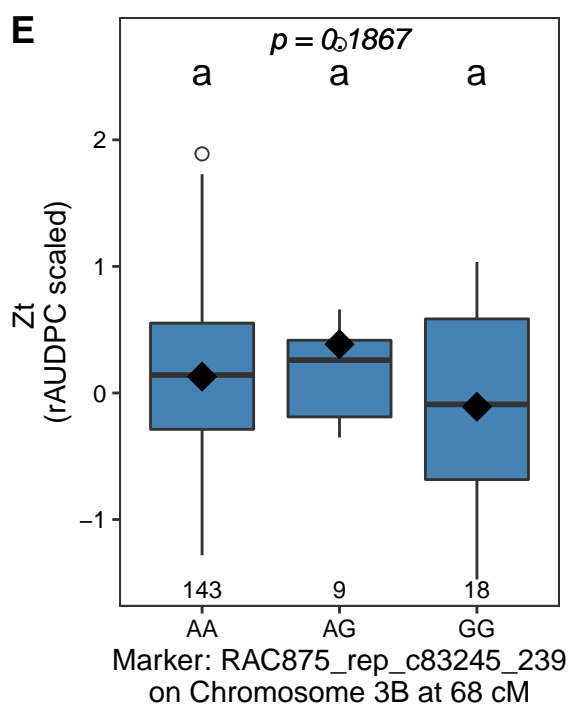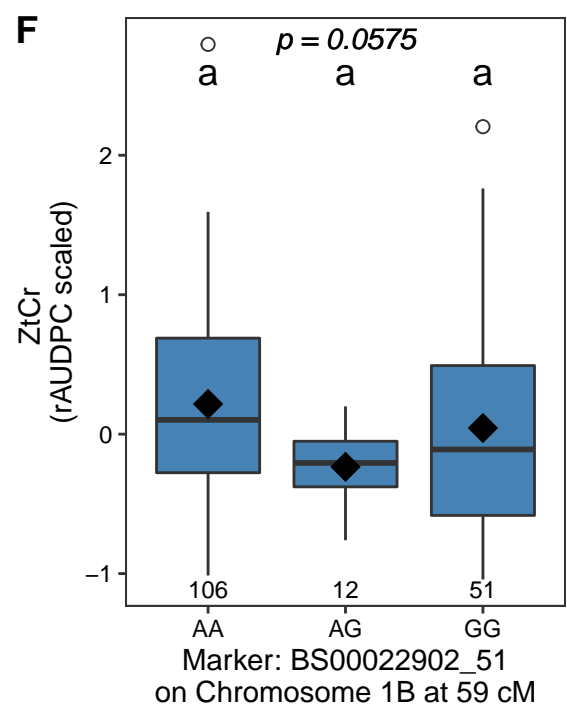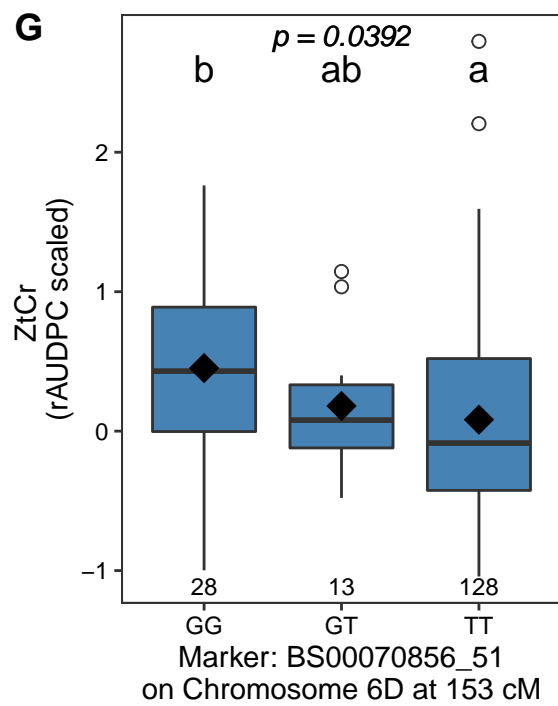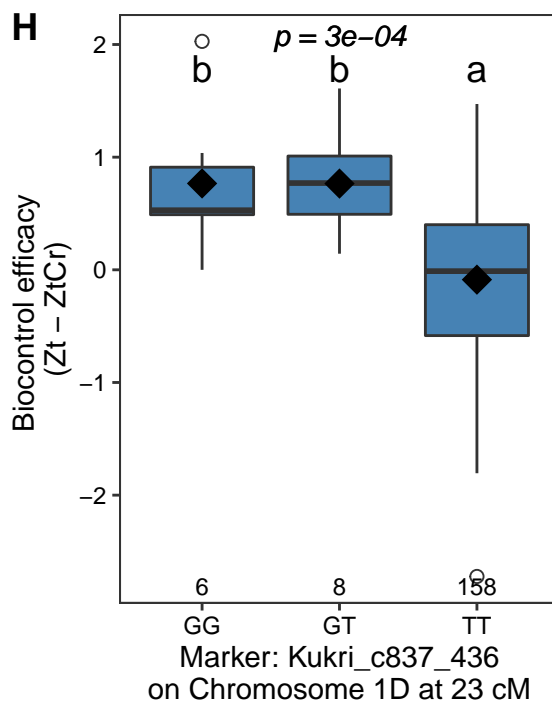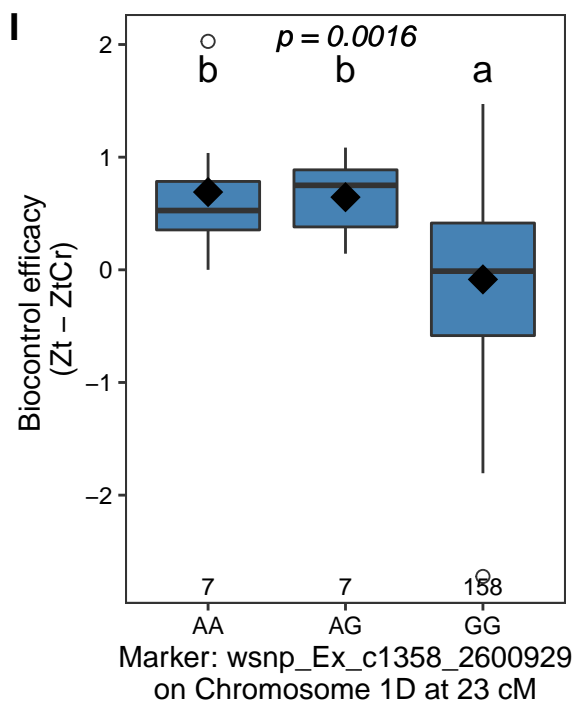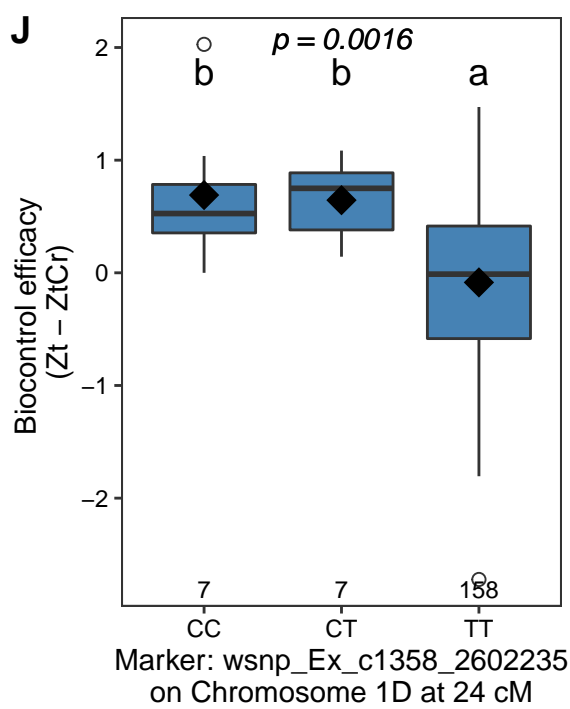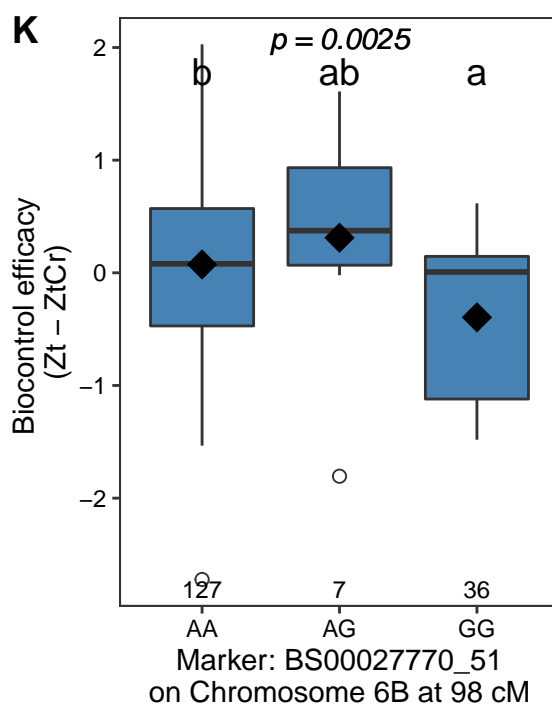

Supplement: Supplementary file 2 — Supplementary Material 2. Supp. Figure 2: Allelic level comparison of SNP markers significantly associated with scaled relative area under disease progress curve (rAUDPC) of wheat genotypes in treatment Zt (Z. tritici alone), treatment ZtCr (Z. tritici along with C. rosea) and biocontrol efficacy (Zt – ZtCr). Panel A-F display significant markers with their location, their alleles and distribution of wheat genotypes for the associated trait. Alleles not sharing the same letter are significantly different at P < 0.05. Numbers at the bottom of the panel indicate number of genotypes, and black diamonds represent mean estimate of the group. [file 12870_2025_6620_MOESM2_ESM.pdf]
